# Editorial: Bone Metastases

**DOI:** 10.3389/fonc.2021.741515

**Published:** 2021-07-30

**Authors:** Maria Teresa Valenti, Monica Mottes, Luca Dalle Carbonare, Olivier Feron

**Affiliations:** ^1^Department of Medicine, University of Verona and Azienda Ospedaliera Universitaria Integrata Verona, Verona, Italy; ^2^Department of Neurosciences, Biomedicine and Movement Sciences, University of Verona, Verona, Italy; ^3^Pole of Pharmacology and Therapeutics (FATH), Institut de Recherche Expérimentale et Clinique (IREC), UCLouvain, Brussels, Belgium

**Keywords:** bone, osteoblasts, osteoclasts, endosteal niche, metastatic niche

Solid tumors often metastasize into bones affecting quality of life and overall survival of cancer patients. Despite improvement of diagnostic tools, the presence of bone metastases often reveals an advanced disease stage with a median survival of a few months and limited appropriate therapies. Moreover, patients with bone metastases suffer from considerable morbidity including pain, fractures and hypercalcemia (1). Bone metastases are often detected in patients with breast, prostate and lung cancers but it is also increasingly recognized that the ability of other types of cancer to form bone lesions has been underestimated for many decades.

Metastasization is a multi-step process where cancer cells escape from the primary tumor, intravasate, survive in the bloodstream and later extravasate from the circulation to develop at a distant site; lymphatics represent another route for migrating cancer cells that will first colonize nodes and later on potentially reach the circulation. Adaptation to the new environment is a pre-requisite for effective growth in conditions different from the primary site. Bone metastases result from complex interactions of cancer cells with hematopoietic stem cells, endothelial cells as well as bone cells (osteoblasts with bone forming activity and osteoclasts with bone resorption activity). In the last years, the contribution of osteoclasts to bone metastasization has been largely investigated including through the dissection of their crosstalk with cancer cells (2). The latter were for instance documented to play a central role in destroying bone upon lowering of extracellular pH, a prerequisite for osteoclast activity (3, 4). Other studies have revealed that cancer cells interact with bone cells either to modulate their dormancy or promote drug resistance (5, 6).

This Research Topic includes two reviews that paint a very broad picture of how the interplay between tumor and bone-resident cells drives the local development of metastases. While Gyori and Moscai review the different osteoclast signaling pathways that are related to the pathological bone loss, Haider et al. describe the interaction between tumor cells and endosteal niche cells during the early stages of breast cancer bone metastasis, with a particular focus on mesenchymal-derived osteoblasts and fibroblasts. The role of calcium, an important building block of bones but also a recognized actor in the development of bone metastases is also addressed in this RT. Through the review of major calcium channels and/or calcium-related routes (ie, TRPs, VGCCs, SOCE, and P2Xs), Yang et al. provide evidence that alterations in calcium homeostasis in bone metastases directly participate in tumor progression. Das et al. also examine the role of calcium by focusing on the Ca^2+^-sensing receptor (CaSR), a dimeric class-C G protein-coupled receptor (GPCR). In their review, these authors explore the hypothesis of CaSR acting as an oncogene in breast cancer and associated bone metastases, facilitating a vicious cycle wherein osteolysis promotes tumor growth and inversely.

Several articles within this RT explore recent developments in the search for therapeutic strategies targeting bone metastases. Kratzsch et al. describe how mTOR inhibitor everolimus but also axitinib, a specific VEGF receptor tyrosine kinase inhibitor, may prevent and retard formation of symptomatic spinal metastases. Interestingly, La Manna et al. who used patient-derived organoids and xenografts (PDX) models from bone-metastatic prostate cancer report the therapeutic benefit of mTORC1/2 inhibitor Rapalink-1, further supporting a role of mTOR pathway in cancers metastasizing in bones. Besides the above anti-oncogenic approaches, targeting metabolism represents another option which may take advantage of the emerging development of new drugs in this field. Tiedemann et al. examine current evidence underlying how altered metabolism in cancer cells may impact on substrate availability for bone cells, and on consecutive alterations in osteoclast differentiation and activity. Xu et al. follow a more focused approach examining how a component of the electron transport chain complex I (ie, NDUFA4L2) accounts for epithelial-to-mesenchymal transition of osteosarcoma cells, pointing out OXPHOS as a critical metabolic path in the development of primary malignant bone tumors. Finally, Xu et al. discuss recent progress on evaluating the role of endoplasmic reticulum stress on bone metastases, identifying a set of potential targets to develop new therapeutic modalities directed against bone metastases. Besides therapeutic approaches, this RT also emphasizes the obvious interest to identify patients at risk for bone metastases in order to treat them at an earlier stage and thereby improve clinical outcomes; current studies evaluating such prognostic biomarkers are summarized by Iuliani et al.

Altogether, the different contributions to this RT offer a series of insightful sets of data to better understand mechanisms involved in the multi-cellular process driving bone metastases and open new perspectives of treatment to counteract the onset of this major life-threatening cancer complication.

## Author Contributions

MV, MM and LD prepared the draft. OF supervised and wrote the paper. All authors contributed to the article and approved the submitted version.

## Conflict of Interest

The authors declare that the research was conducted in the absence of any commercial or financial relationships that could be construed as a potential conflict of interest.

## Publisher’s Note

All claims expressed in this article are solely those of the authors and do not necessarily represent those of their affiliated organizations, or those of the publisher, the editors and the reviewers. Any product that may be evaluated in this article, or claim that may be made by its manufacturer, is not guaranteed or endorsed by the publisher.
